# Supplementary figures and images for: Jellyfish extract induces apoptotic cell death through the p38 pathway and cell cycle arrest in chronic myelogenous leukemia K562 cells
Source: PeerJ. 2017 Jan 19;5:e2895. doi: 10.7717/peerj.2895 (PMC5251936; doi:10.7717/peerj.2895)

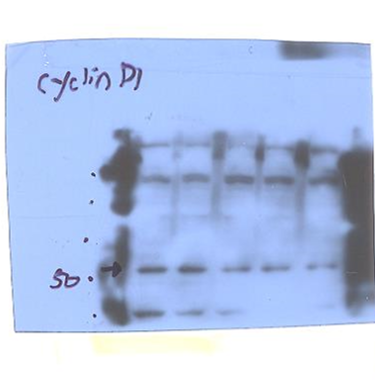

Supplement: Data S1 [file peerj-05-2895-s001.zip › Figure 6. C) cyclinD1.jpg.png]

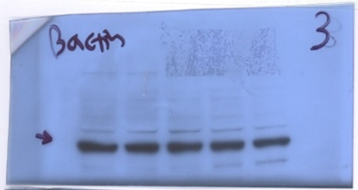

Supplement: Data S1 [file peerj-05-2895-s001.zip › Figure 4. A) beta actin.png]

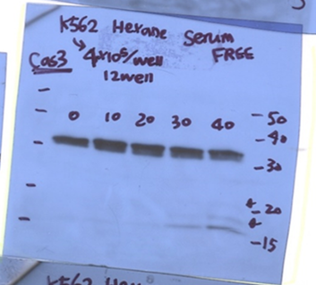

Supplement: Data S1 [file peerj-05-2895-s001.zip › Figure 4. A) caspase-3.png]

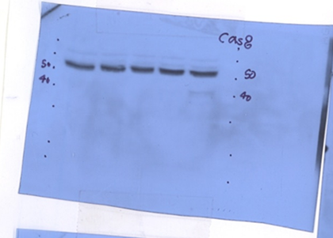

Supplement: Data S1 [file peerj-05-2895-s001.zip › Figure 4. A) caspase-8.png]

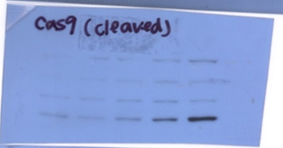

Supplement: Data S1 [file peerj-05-2895-s001.zip › Figure 4. A) caspase-9.png]

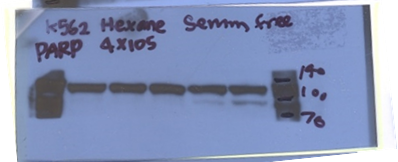

Supplement: Data S1 [file peerj-05-2895-s001.zip › Figure 4. A) PARP.png]

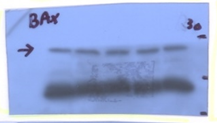

Supplement: Data S1 [file peerj-05-2895-s001.zip › Figure 4. C) BAX.png]

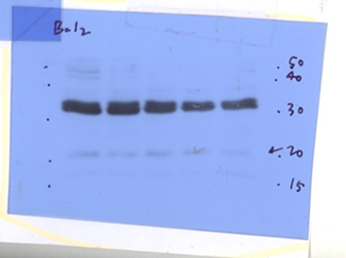

Supplement: Data S1 [file peerj-05-2895-s001.zip › Figure 4. C) Bcl2.png]

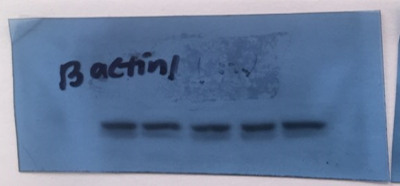

Supplement: Data S1 [file peerj-05-2895-s001.zip › Figure 4. C) beta actin.png]

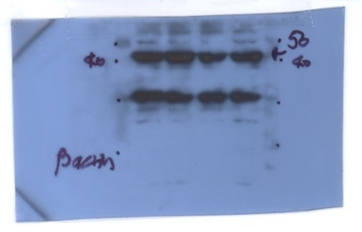

Supplement: Data S1 [file peerj-05-2895-s001.zip › Figure 4. F) beta actin.png]

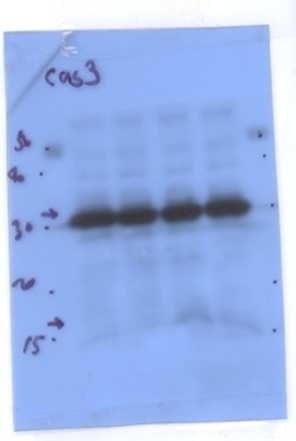

Supplement: Data S1 [file peerj-05-2895-s001.zip › Figure 4. F) caspase-3.png]

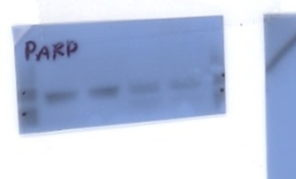

Supplement: Data S1 [file peerj-05-2895-s001.zip › Figure 4. F) PARP.png]

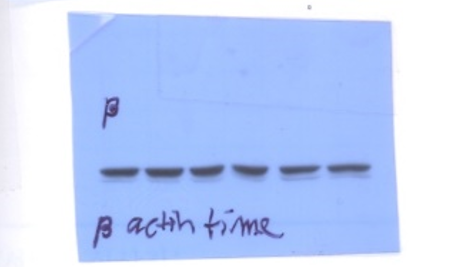

Supplement: Data S1 [file peerj-05-2895-s001.zip › Figure 5. A) beta actin.png]

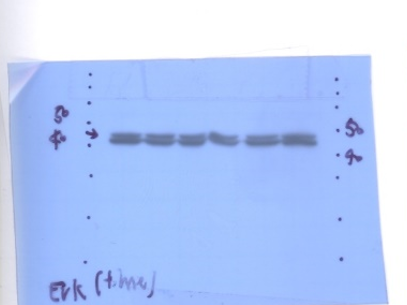

Supplement: Data S1 [file peerj-05-2895-s001.zip › Figure 5. A) ERK.png]

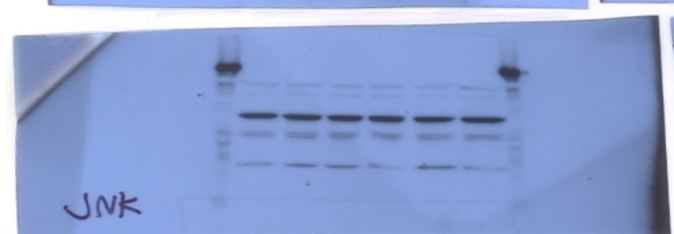

Supplement: Data S1 [file peerj-05-2895-s001.zip › Figure 5. A) JNK.png]

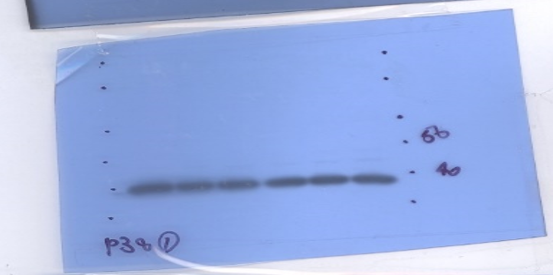

Supplement: Data S1 [file peerj-05-2895-s001.zip › Figure 5. A) p38.png]

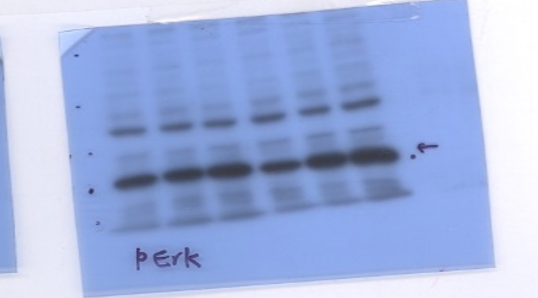

Supplement: Data S1 [file peerj-05-2895-s001.zip › Figure 5. A) p-ERK.png]

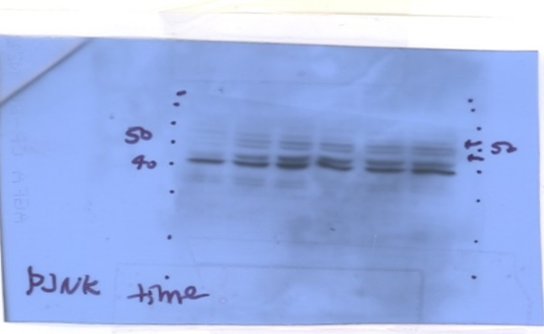

Supplement: Data S1 [file peerj-05-2895-s001.zip › Figure 5. A) p-JNK.png]

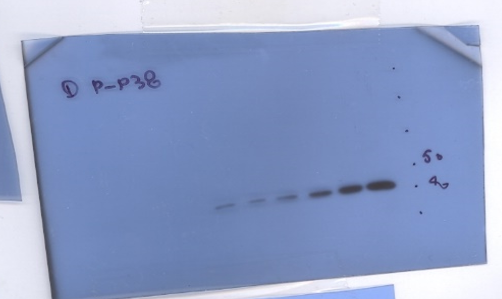

Supplement: Data S1 [file peerj-05-2895-s001.zip › Figure 5. A) p-p38.png]

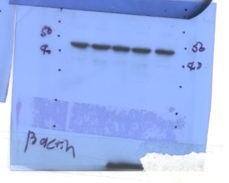

Supplement: Data S1 [file peerj-05-2895-s001.zip › Figure 5. C) beta actin.png]

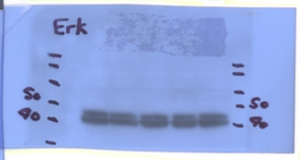

Supplement: Data S1 [file peerj-05-2895-s001.zip › Figure 5. C) ERK.png]

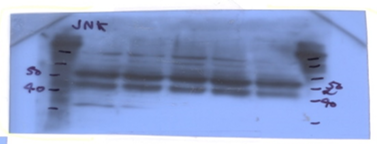

Supplement: Data S1 [file peerj-05-2895-s001.zip › Figure 5. C) JNK.png]

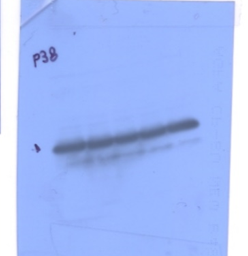

Supplement: Data S1 [file peerj-05-2895-s001.zip › Figure 5. C) p38.png]

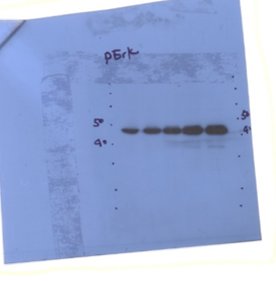

Supplement: Data S1 [file peerj-05-2895-s001.zip › Figure 5. C) p-ERK.png]

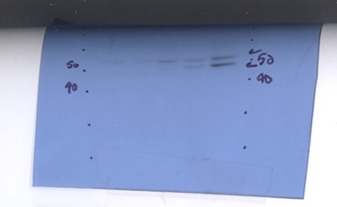

Supplement: Data S1 [file peerj-05-2895-s001.zip › Figure 5. C) p-JNK.jpg.png]

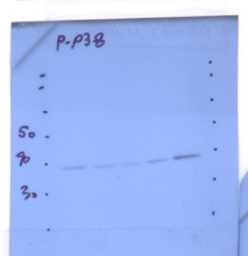

Supplement: Data S1 [file peerj-05-2895-s001.zip › Figure 5. C) p-p38.png]

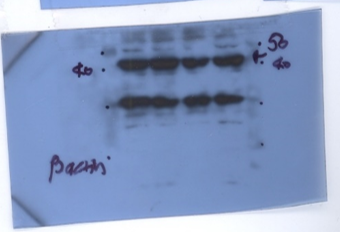

Supplement: Data S1 [file peerj-05-2895-s001.zip › Figure 5. F) beta actin.png]

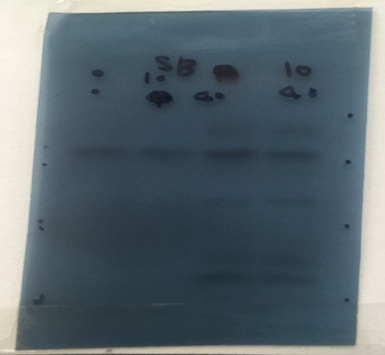

Supplement: Data S1 [file peerj-05-2895-s001.zip › Figure 5. F) caspase-3.png]

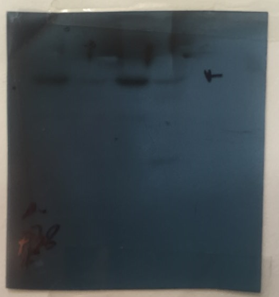

Supplement: Data S1 [file peerj-05-2895-s001.zip › Figure 5. F) p-p38.png]

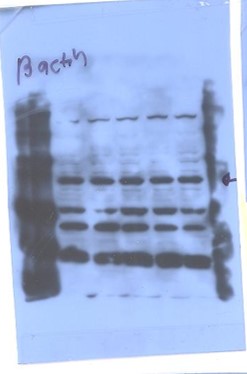

Supplement: Data S1 [file peerj-05-2895-s001.zip › Figure 6. C) beta actin.jpg]

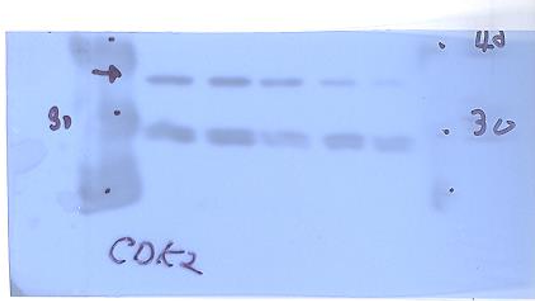

Supplement: Data S1 [file peerj-05-2895-s001.zip › Figure 6. C) CDK2.png]

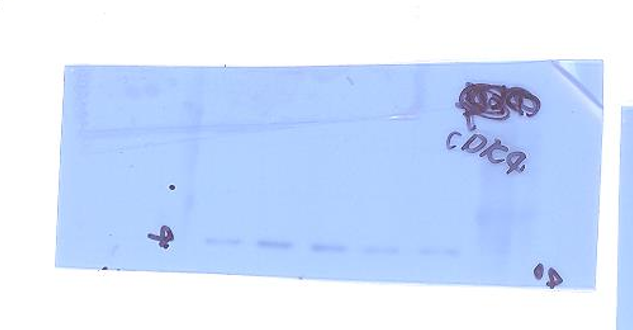

Supplement: Data S1 [file peerj-05-2895-s001.zip › Figure 6. C) CDK4.png]

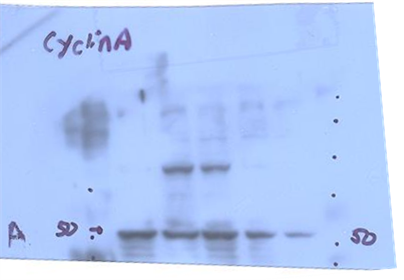

Supplement: Data S1 [file peerj-05-2895-s001.zip › Figure 6. C) cyclinA.png]

Supplementary Figure 1.

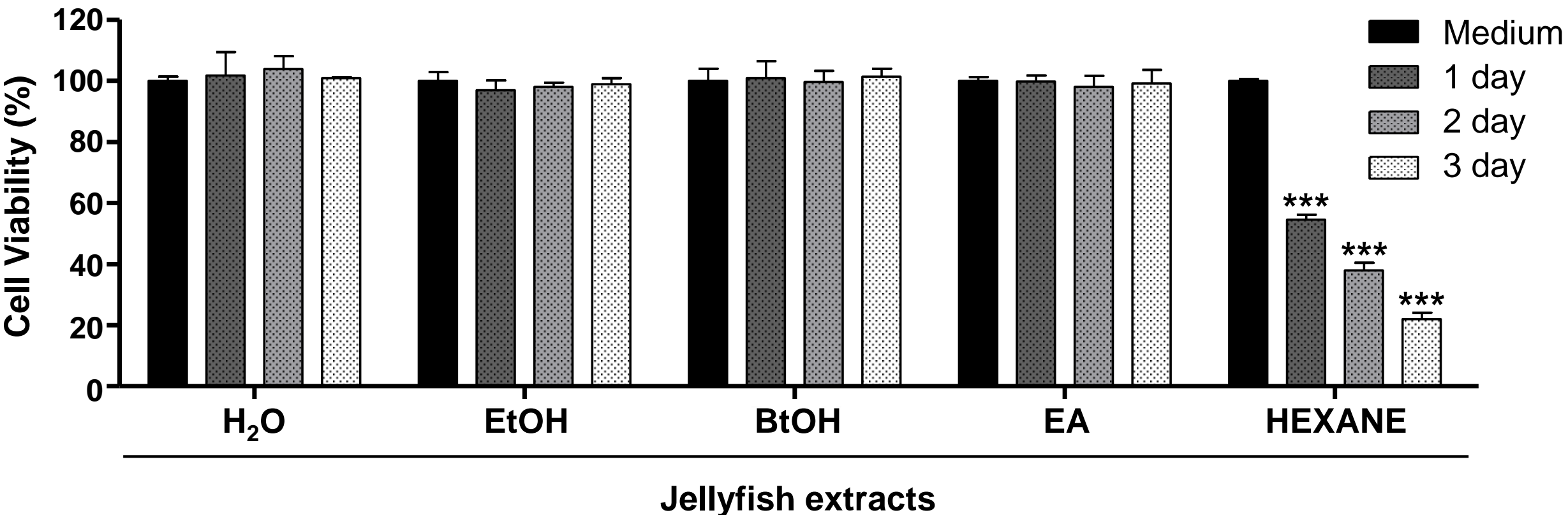

Supplement: Figure S1 — K562 cells were separately treated with H2O extracts, EtOH extracts, BuOH extracts, EA extracts and hexane extracts for 1 to 3 days and cell viabilities were measured using MTT assay. *** P < 0.0005 vs. medium (untreated). [file peerj-05-2895-s005.pdf]

Supplementary Figure 2.

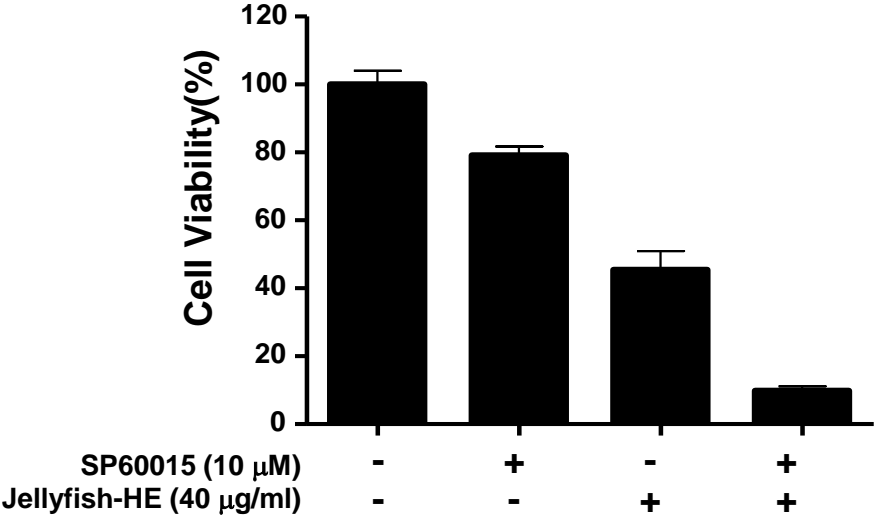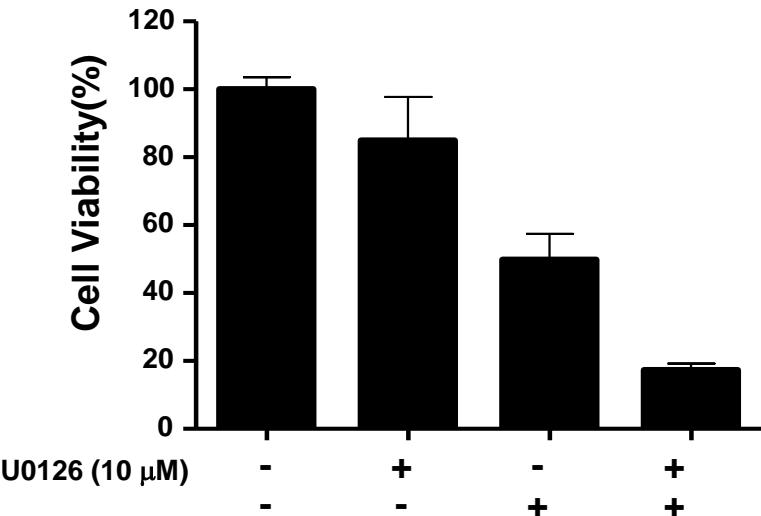

Supplement: Figure S2 — After treatment of the cells with 40 µg/ml Jellyfish-HE in the presence or absence of JNK inhibitor SP60015 (A) or ERK inhibitor U0126 (B), cell viability was measured by the MTT assay. [file peerj-05-2895-s006.pdf]
